# Humidity-Insensitive NO_2_ Sensors Based on SnO_2_/rGO Composites

**DOI:** 10.3389/fchem.2021.681313

**Published:** 2021-05-28

**Authors:** Yingyi Wang, Lin Liu, Fuqin Sun, Tie Li, Ting Zhang, Sujie Qin

**Affiliations:** ^1^ Department of Health and Environmental Sciences, Xi’an Jiaotong-Liverpool University, Suzhou, China; ^2^ I-Lab, Key Laboratory of Multifunctional Nanomaterials and Smart Systems, Suzhou Institute of Nano-Tech and Nano-Bionics (SINANO), Chinese Academy of Sciences (CAS), Suzhou, China; ^3^ School of Nano-Tech and Nano-Bionics, University of Science and Technology of China, Hefei, China

**Keywords:** gas sensor, SnO_2_/rGO composites, humidity-insensitive, low temperature, nitrogen dioxide (NO_2_)

## Abstract

This study reported a novel humidity-insensitive nitrogen dioxide (NO_2_) gas sensor based on tin dioxide (SnO_2_)/reduced graphene oxide (rGO) composites through the sol-gel method. The sensor demonstrated ppb-level NO_2_ detection in p-type sensing behaviors (13.6% response to 750 ppb). Because of the synergistic effect on SnO_2_/rGO p-n heterojunction, the sensing performance was greatly enhanced compared to that of bare rGO. The limit of detection of sensors was as low as 6.7 ppb under dry air. Moreover, benefited from the formed superhydrophobic structure of the SnO_2_/rGO composites (contact angle: 149.0°), the humidity showed a negligible influence on the dynamic response (S_g_) of the sensor to different concentration of NO_2_ when increasing the relative humidity (RH) from 0 to 70% at 116°C. The relative conductivity of the sensor to 83% relative humidity was 0.11%. In addition, the response ratio (S_g_/S_RH_) between 750 ppb NO_2_ and 83% RH was 649.0, indicating the negligible impaction of high-level ambient humidity on the sensor. The as-fabricated humidity-insensitive gas sensor can promise NO_2_ detection in real-world applications such as safety alarm, chemical engineering, and so on.

## Introduction

Air pollution has become a pressing global issue facing the development of industry and technology. Nitrogen dioxide (NO_2_), as one of the most toxic air pollutants, is generated from industries and vehicle emissions, which can cause some serious environmental issues such as haze, photochemical smog, and acid rain (Mallik and Lal, 2014). United Statess Occupational Safety and Health Administration (OSHA) reported that when the concentration of NO_2_ is over 1 ppm, inhalation of NO_2_ for 15 min will cause some respiratory diseases such as asthma (Cheng, et al., 2019; Yuvaraja, et al., 2020). In the urban atmosphere, the concentration of NO_2_ commonly measured in the range from 20 to 100 ppb (Brunet, et al., 2008). Therefore, a NO_2_ sensor, which is sensitive enough to detect at least 20 ppb or ultralow concentration of NO_2_ (ppb level), is urgently demanded.

Up to now, metal oxide semiconductors-based NO_2_ sensors have been used in many applications owing to high chemical stability and low cost of sensing materials such as CuO (Bo, et al., 2020; Wang Y, et al., 2020), ZnO (Wang J, et al., 2019; Choi, et al., 2020), WO_3_ (Liu, et al., 2020; Wang M, et al., 2020), NiO (Wei, et al., 2019; Wilson, et al., 2020), and SnO_2_ (Kamble, et al., 2017; Zhong, et al., 2019). Among them, SnO_2_ is a typical n-type wide bandgap semiconductor (3.6 eV) that has been regarded as one of the most promising industrialized candidates for NO_2_ sensing due to its attractive characteristics, including controlled size, low limits of detection, and facile large-scale fabrication (Maeng, et al., 2014; Lee, et al., 2015). However, there are several shortcomings of pure SnO_2_-based sensors that limit their practical applications. Firstly, aggregation occurs when the size is too small during the material synthesis and the sensor fabrication processes, which decreases the surface-specific area, reduces the sensitivity of gas sensors and influences the long-term stability of the as-fabricated sensor (Li, et al., 2015; Wu, et al., 2020). Meanwhile, the SnO_2_-based gas sensor generally needs a high operation temperature (>200°C) to achieve high sensitivity, which causes high power consumption (Van Hieu, 2010; Guo J, et al., 2016). Nowadays, combining SnO_2_ with low-dimensional materials (e.g., MoS_2_ (Qiao, et al., 2018), carbon nanotube (Minh Nguyet, et al., 2017), metal oxide (Park, et al., 2020), graphene (Hu, et al., 2020), etc.) to form p-n heterojunction has been regarded as the prospective strategies to overcome the shortcomings of pure SnO_2_-based gas sensors (Yin, et al., 2014; Gui, et al., 2018). Among low-dimensional materials, owing to the low-cost, ultrahigh specific surface area, controllable bandgap and various oxygen-containing functional group (e.g., C-O, O-C=O, and O-C(O)-O), rGO is an ideal material to obtain high-performance gas sensor by decorating with metal oxides (Guo, et al., 2019; Yin, et al., 2020). Hence, constructing p-n heterojunction between rGO and SnO_2_ is a potential approach to enhance the sensing performance of sensors through modulating the carrier transportation due to different working functions (Neri, et al., 2013; Choi, et al., 2014; Kim, et al., 2018). For example, Zhu, et al. (2017) reported a NO_2_ gas sensor based on rGO/SnO_2_ nanocomposites, and the sensing response of sensors increased three times compared with that of sensors based on pure rGO. Meanwhile, Kim, et al. (2017) fabricated graphene-SnO_2_ nanocomposites (SnO_2_-G)-based sensors, and the sensing response of SnO_2_-G-based sensors toward 1 ppm NO_2_ is 24.7, which is around two folds higher than that of SnO_2_-based sensors. Similarly, Lin, et al. (2020) prepared Co_3_O_4_/N-doped rGO (N-rGO) nanocomposites-based ethanol sensors. The sensing response value of Co_3_O_4_/N-rGO-based sensors to 100 ppm ethanol at 200°C is around 20 folds higher than that of N-rGO-based sensors.

Although heterojunction enhances the gas sensing performance, the high-level nonconstant humidity will severely impact the sensing performances of these reported NO_2_ sensors, which is difficult to distinguish the target gas from ambient humidity and hinder the applications in the real world. For example, Wang, et al. (2018) fabricated Pd-SnO_2_-RGO-based NO_2_ sensors and showed 76% sensing response to 1 ppm NO_2_ gas. However, when the sensor exposed to NO_2_ gas with 80% RH, the sensing response declined by 20%. The limit of detection of Pd-SnO_2_-RGO-based NO_2_ sensors is 50 ppb under dry air. Similarly, Guo, et al. (2019) reported Bi@rGO/SnO_2_-based benzene sensors. The sensing response to 5 ppm benzene with 60% RH is three times lower than that without humidity. Degler, et al. (2018) found that the gas sensing response cannot be controlled by doping different noble metal content (Pt) on SnO_2_. To date, there are some strategies have been developed to address this issue, including the construction of gas preconcentration techniques and dehumidification techniques (Groves and Zellers, 2001; Peng, et al., 2008). However, these methods are costly, complex, and sacrifice the sensor’s sensitivity (Konvalina and Haick, 2012).

To solve these problems, we proposed the humidity-insensitive NO_2_ sensors based on SnO_2_/rGO p-n heterojunction, which was used to detect NO_2_ at low temperatures (as low as 116°C). The sensing performance of SnO_2_/rGO composite-based NO_2_ sensors was studied, and the LOD was found to be as low as 6.7 ppb, which is below the standard of United States OSHA and NO_2_ pollutant concentration in the urban atmosphere. Notably, the NO_2_ sensor showed a reliable sensing response under increasing relative humidity conditions (3–70% RH), and the resistance of the sensor almost kept constant under 83% RH, promising real-world applications.

## Materials and Methods

### Materials and Synthesis of Composites

Natural graphite flake (325 meshes, 99.8%) was purchased from Sigma Aldrich; H_2_SO_4_ (AR) and KMnO_4_ (AR) was purchased from Shanghai Hushi Laboratorial Equipment Co., Ltd.; SnCl_4_.5H_2_O (AR) was purchased from Macklin; Epichlorohydrin (PPD) (Analytical reagent) and N, N-Dimethylformamide (DMF) (97%) were purchased from Aladdin. These reagents were used without any further purification. The micro-hotplates were purchased from Leanstar-Tech Co., Ltd. The SnO_2_/rGO composite was prepared through a sol-gel method. Briefly, the GO was synthesized from nature graphite powder based on modified Hummer’s method. Secondly, the SnCl_4_·5H_2_O and epichlorohydrin (PPD) were slowly added into the GO/DMF solution and stirred for a short time. The SnO_2_/rGO composite was formed after three days of solution exchange and dried by supercritical CO_2_ to carbonize at 600°C for 2 h under the Ar atmosphere. The synthesized sensing material was suspended in a dimethylformamide solution (DMF) solution. The SnO_2_/rGO composite was drop-coated onto the micro-hotplate to fabricate gas sensors. After that, sensors were annealed at 200°C for 20 min under Ar atmosphere protection to reduce the contacting barrier.

### Characterization of Composites and Microstructures

The morphology and crystal structure of SnO_2_/rGO composites were analyzed by scanning electron microscopy (SEM, Hitachi-s4800), transmission electron microscopy (TEM, Tecnai G2 F20 S-Twin). The chemical composite of materials was carried out by energy dispersive spectrometry (EDS, FEI, Quanta FEG 250). The crystal lattice was analyzed by X-ray Diffraction (XRD, Bruker AXS, D8 Advance). The degree of reduction of rGO was characterized by Raman spectroscopy (Raman, Horriba-JY, LABRAM HR).

### Measurements Sensing Performance of NO_2_ Sensors

The gas sensing performance under dry air (∼3% RH) was investigated by a designed testing system based on previous work (Liu, et al., 2019). In general, firstly, the sensor chip was connected in series with a loaded resistor, which was selected to close to sensor resistance to optimize the resolution obtained from measurements. Secondly, the specific concentration of NO_2_, which was implemented by dry air (80% N_2_ and 20% O_2_) and controlled by mass flow controllers (MFCs, Sevenstar CS200, China), goes through a quartz chamber (volume: 1 cm^3^, 200 sccm). Finally, sensor resistance was determined by the Fieldpoint analog input and output modules by continuously controlling and monitoring the voltage of the circuit (National Instruments, Austin, TX). The loaded resistor calculated by Ohm’s law was recorded in a custom LabView computer program (Supplementary Figure S1).

### Measurements Sensing Performance of NO_2_ Sensors Under Humidity

Dynamic conductivity response to high humidity conditions (83% RH) was tested by the KNO_3_ saturated saline solution, added to a humidity controller (Supplementary Figure S1). Saturated saline solutions produce various saturated vapor pressures and form different relative humidity (Greenspan, 1977). One commercial high-precision temperature/humidity sensor (Sensirion Company, SHT75) was used as the reference sensor to detect real-time humidity. The static relative response to various concentrations of NO_2_ under 70% RH was tested in the homemade testing chamber (20 L) (Supplementary Figure S2). The specific high concentration of NO_2_ gas was injected into the testing chamber and diluted by the air in the testing chamber. The air humidity (∼70% RH) of the day is recorded as the ambient humidity.

## Results and Discussion

### Material Characterizations

**FIGURE 1:**
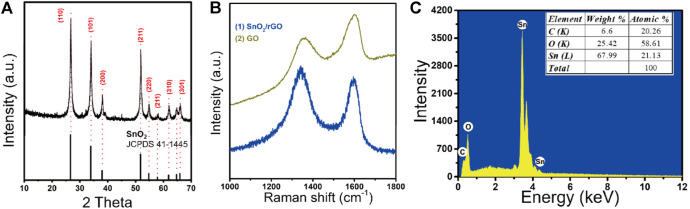
**(A)** The XRD patterns of SnO_2_/rGO composites; **(B)** the Raman spectra of SnO_2_/rGO composites and GO; **(C)** the EDS analysis of SnO_2_/rGO composites.

The crystal structure of SnO_2_/rGO composites was examined by XRD, as shown in Figure 1A. The positions of characteristic peaks are located at 2θ = 26.61°, 33.89°, 37.95°, 51.78°, 54.76°, 64.72°, and 65.94°, which are, respectively, collaborated with (110), (101), (200), (211), (220), (310), and (301) planes of tetragonal rutile SnO_2_ (JCPDS. 41–1,445). Compared with the reported XRD patterns of pristine rGO at 2θ = 24.7° and 42.8° (Wang Z, et al., 2019), SnO_2_/rGO composites do not show a peak assigned to rGO. The high density of SnO_2_ nanoparticles is uniformly decorated on the surface of the rGO, which can prevent the reassembled behavior of SnO_2_ and cover its XRD pattern information (Zhang, et al., 2014). Moreover, the Raman spectroscopy was employed to further study the reduced structure of the rGO in SnO_2_/rGO composites (Figure 1B). The peaks located at around 1,341 and 1,594 cm^−1^ are assigned to the typical D band and G band of rGO, respectively, (Guo D, et al., 2016). The intensity ratio of D peak to G peak (I_D_/I_G_) of SnO_2_/rGO (1.12) is higher than that of GO (0.81), which indicates that the oxygen functional groups (e.g., C-O, O-C=O, and O-C(O)-O) have been removed and induced defects during the synthesis process (Tuan, et al., 2018; Hu, et al., 2017; Xu, et al., 2019). The EDS analysis (Figure 1C) shows that the product contained C, O, Sn elements. The atomic ratios of Sn, O, and C are 21.13, 58.61, and 20.26%, respectively. These results indicate that no other impurities and crystals were mixed in the reaction product.

**FIGURE 2:**
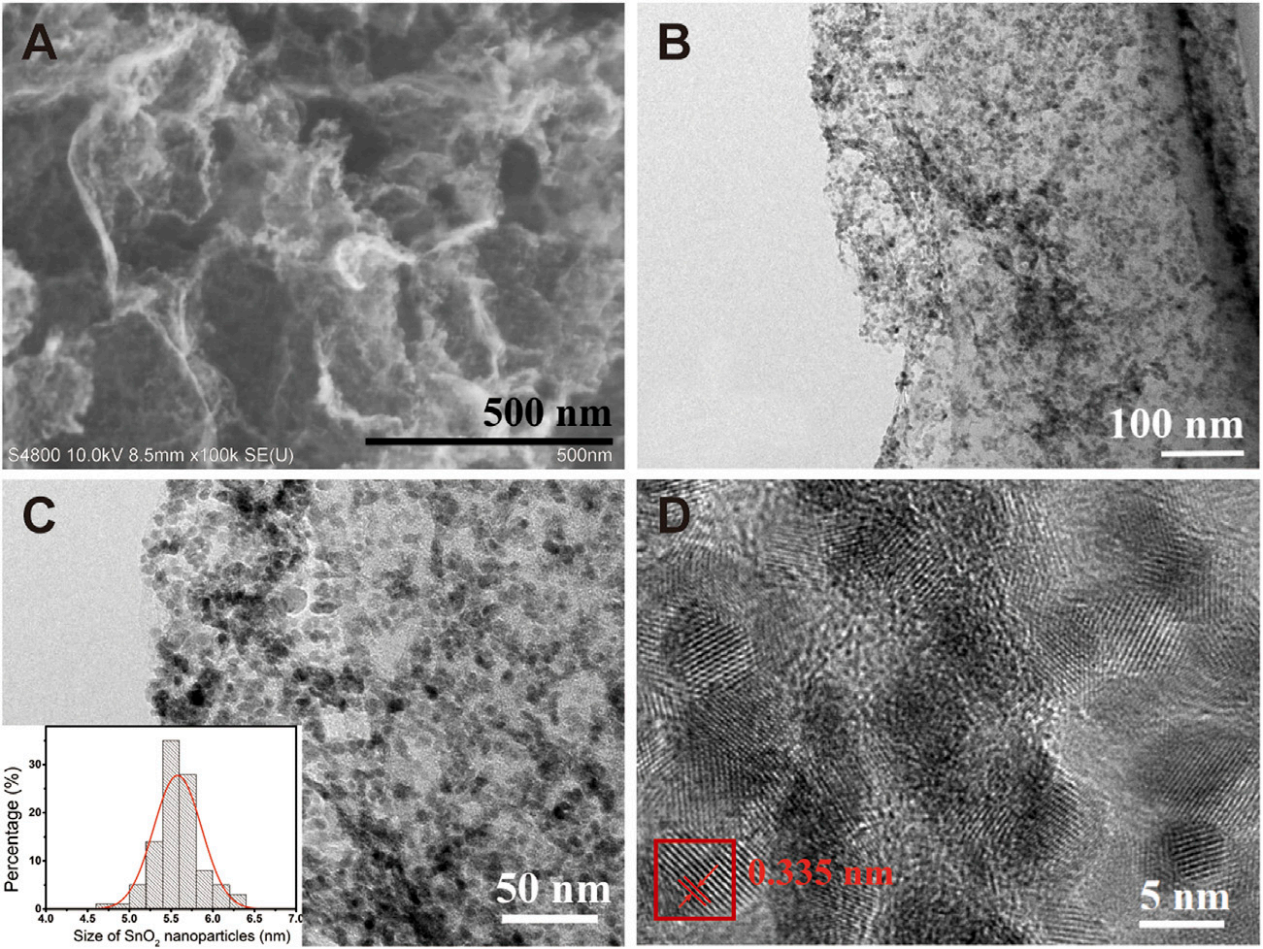
**(A)** Typical SEM images of SnO_2_/rGO composites; **(B, C)** Low magnification TEM images of SnO_2_/rGO composites, (c, inset) SnO_2_ NPs size distribution histogram; **(D)** the HRTEM image and corresponding crystalline interplanar of SnO_2_/rGO composites.

The morphology of SnO_2_/rGO composites was characterized via the SEM and TEM techniques and displayed in Figure 2. It can be seen from Figure 2A,B that the SnO_2_ nanoparticles (NPs) are uniformly and densely anchored on the surfaces of the rGO nanosheets without any agglomeration. The low-resolution TEM images in Figure 2C show the size distribution of the SnO_2_ NPs in the composites, around 5.5 nm with normal distribution from 4.5 to 6.5 nm (Figure 2C, inset). Moreover, the high-resolution TEM (HRTEM) image of SnO_2_/rGO in Figure 2D shows that the SnO_2_ NPs are highly crystallized with a crystalline interplanar spacing of ∼0.335 nm, which is attributed to the (110) crystal plane.

### Gas Sensing Performances

**FIGURE 3:**
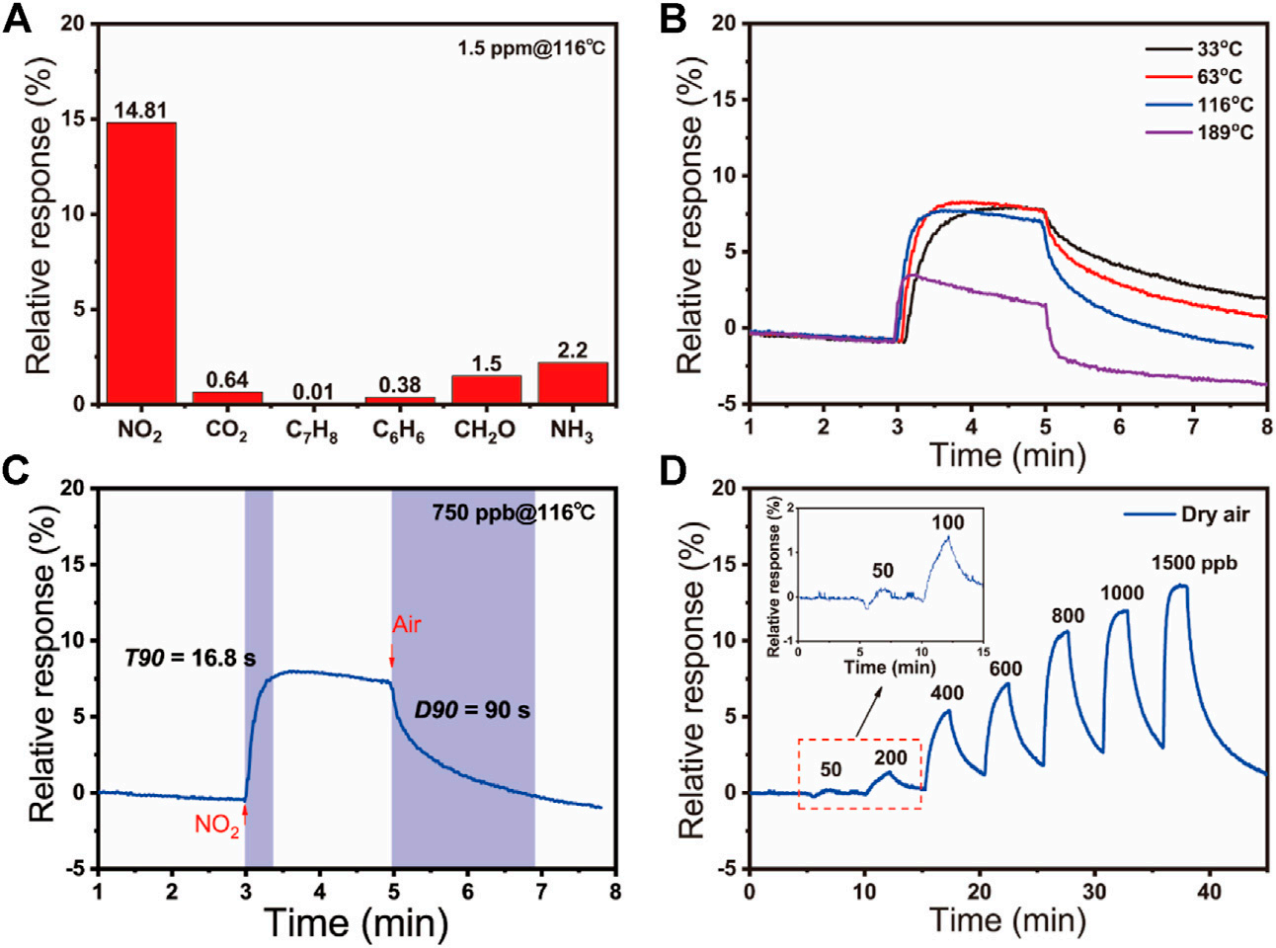
**(A)** The relative responses of SnO_2_/rGO composite-based sensors to different gases (NO_2_, CO_2_, C_7_H_8_, C_6_H_6_, CH_2_O, and NH_3_) at 116°C; **(B)** The response curves to 750 ppb NO_2_ at the increasing operating temperature of 33, 63, 116, 189°C; **(C)** The response curve to 750 ppb NO_2_ of sensors based on SnO_2_/rGO composites at 116°C; **(D)** The relative responses to different concentrations of NO_2_ (50–1500 ppb) under dry air.

The gas sensing performance of the SnO_2_/rGO composite-based sensors was tested. The relative responses of the sensors were defined as the relative changes of resistance in the air and those in target gases: S_g_ = (|R_g_−R_a_|/R_a_)*100% for oxidizing gas or S_g_ = (|R_a_−R_g_|/R_a_)*100% for reducing gas (where R_a_ is the sensor resistance in air and R_g_ is the sensor resistance in target gas). Figure 3A shows the response of these sensors to 1.5 ppm of various gases at 116°C, including ammonia (NH_3_), methanal (CH_2_O), benzene (C_6_H_6_), toluene (C_7_H_8_), carbon dioxide (CO_2_), and NO_2_. The result reveals that the as-prepared SnO_2_/rGO composite has high selectivity to NO_2_ compared with other gases. It attributed to the relatively high adsorption energy of NO_2_ on sensing materials among these gases (Huang, et al., 2008; Schröder, 2013; Lazar, et al., 2013; Chakradhar, et al., 2016; Zhu, et al., 2021), indicating its high selectivity to NO_2_ (Supplementary Table S1). To determine the optimized operating temperature, Figure 3B and Supplementary Table S1 show the response plots of SnO_2_/rGO composite-based NO_2_ sensors toward 750 ppb NO_2_ at the serial operation temperature from 33 to 189°C. The relative response of sensors at low temperature (e.g., 33, 63, and 116°C) have slight difference (7.80% at 33°C, 8.29% at 63°C, and 7.57% at 116°C) and then it dramatically decreased by further increasing the operation temperature (3.51% at 189°C). However, the response time (T90) and recovery time (D90) dramatically decrease along with the operating temperature increase. The T90 and D90 are, respectively, defined as the time required for a sensor to reach 90% of the stable resistance value when the test gas is turned on and off. As Figure 3C shows, the fast T90 (7 s) and D90 (31 s) of NO_2_ sensors to 750 ppb NO_2_ are obtained when the operating temperature was elevating to 189°C. Decreasing the operating temperature to 116°C, the T90 and D90 of NO_2_ sensors increase to 17 and 90 s, respectively, (Figure 3C; Supplementary Figure S3). When the devices worked at 63 and 33°C, the T90 and D90 have been further increased (Supplementary Figure S4; Supplementary Table S2). Because of the similar and relatively high response value at 33, 63, and 116°C, SnO_2_/rGO composite-based sensors are regarded as the candidate to work at room temperature. The temperature can affect the adsorption/desorption process on the sensing materials and sensor surface. The rate of adsorption/desorption increases as the temperature rose, resulting in a shorter T90 and D90 (Walker, et al., 2019). However, when the temperature arrives too high, the quantity and properties of active sites on the surface of sensing materials have been changed (Neri, et al., 2013), which causes the adsorbed oxygen species and NO_2_ cannot remain on the surface of sensing materials to undergo a reaction (Jinkawa, et al., 2000), resulting in a low sensing response and the drifting of baseline were observed correspondently. Therefore, taking the T90, D90, and the relative response of NO_2_ sensors into consideration, the optimal operating temperature was chosen to be 116°C. Figure 3D demonstrates the dynamic response of sensors to various NO_2_ concentrations at dry air from 50 to 1,500 ppb, indicating a broad response range. Contrastively, the sensing performances of pure rGO to 4 ppm were dramatically declined (Supplementary Figure S5), which attracted the electrons from rGO to SnO_2_ and enhanced sensing performance by SnO_2_/rGO composite-based NO_2_ sensors.

**FIGURE 4:**
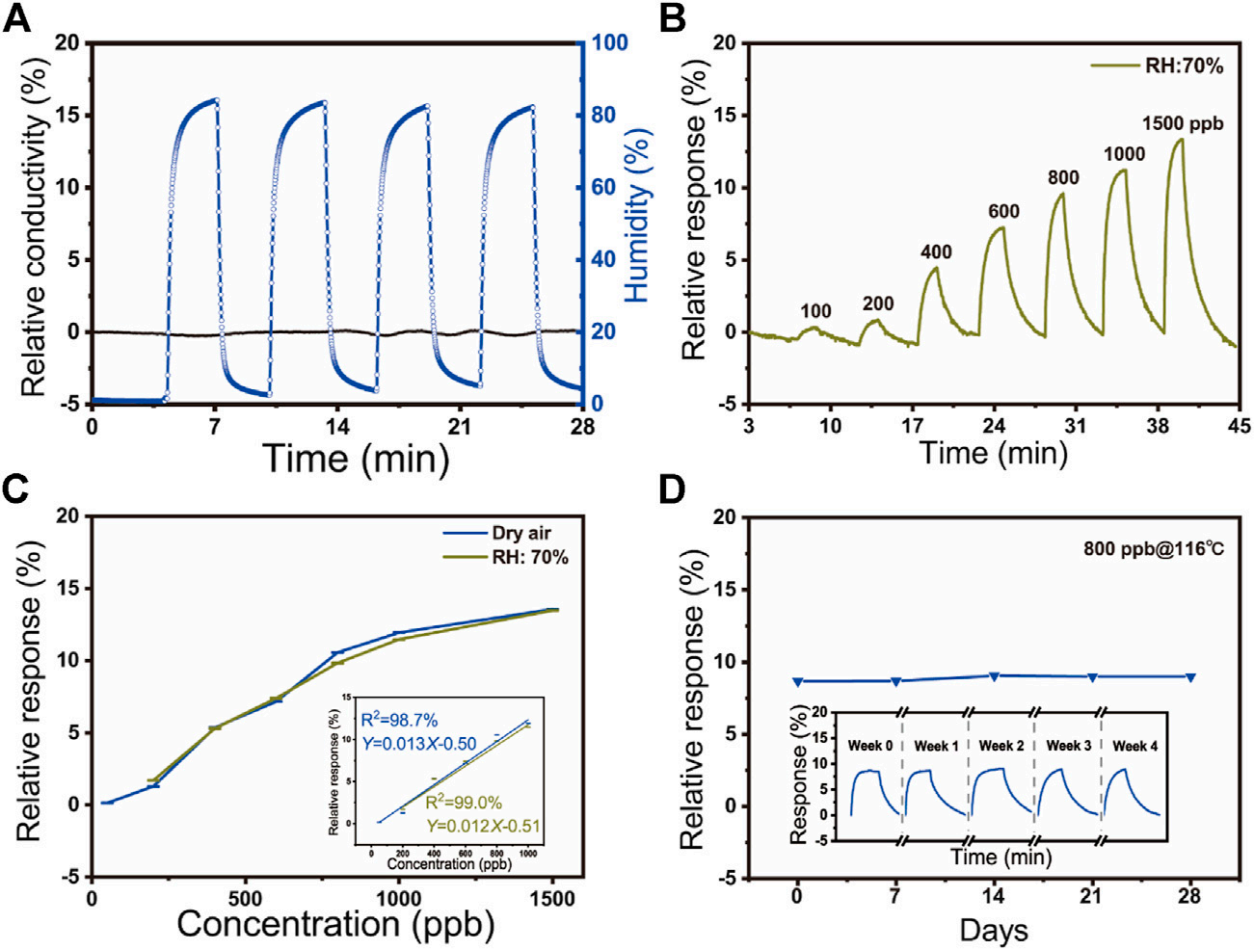
**(A)** The relative response of sensors in high humidity in four cycles (83% RH); **(B)** The relative response to different concentrations of NO_2_ (50–1500 ppb) under 70% RH environment at 116°C; **(C)** The relative response of the various NO_2_ concentration to ambient humidity at 116°C; (c. inset) The linear fit between the concentration of NO_2_ and dynamic response under dry air (blue) and 70% RH (green) at 116°C; **(D)** Long time stability of SnO_2_/rGO composite-based sensors to 800 ppb NO_2_ at room temperature for about 28 days; (d. inset) The repeatability of sensors to 800 ppb NO_2_ during the five testing cycles at 116°C.

The dynamic responses of SnO_2_/rGO composite-based NO_2_ sensors toward high humidity (around 83% RH) have been measured (Figure 4A). It can be seen that the relative conductivity of NO_2_ sensors shows extremely weak fluctuation (∼0.11%). Compared with other works and the commercial bare NO_2_ sensor (MEMS NO_2_ sensor, GM-102B, Zhenzhou Winsen Electronics Technology Co., Ltd.) summarized in Supplementary Table S3, as-fabricated SnO_2_/rGO composite-based NO_2_ sensors showed an extremely high response ratio (S_g_/S_RH_ = 649.0) between 750 ppb NO_2_ and 83% RH, which indicates that the high-level ambient humidity shows negligible impaction on the NO_2_ sensor. Moreover, the static sensing performance of the sensor to different concentrations of NO_2_ (from 200 to 1,500 ppb) under the real-world environment (70% RH) was estimated (Figure 4B). Compared with the above-obtained results under dry air, as Figure 4C showed, the two curves have similar trends and the effect of humidity on the sensor can be neglected. The humidity-insensitive property of high-performance NO_2_ sensors attributes to the formed superhydrophobic structure of SnO_2_/rGO composites, which the exhibited contact angle is 149.0° (Supplementary Figure S6). Two main factors determine the superhydrophobicity of a material surface: surface roughness and surface energy. In general, a rough surface with low surface energy leads to a hydrophobic surface (Lin, et al., 2011; Chen and Dong, 2013). The as-fabricated SnO_2_/rGO composites by this sol-gel method have high porosity and high surface roughness (Lin, et al., 2011). Meanwhile, the high anneal temperature (600°C) during carbonized process vastly decreases the hydrophilic oxygen-containing function groups on the rGO surface (e.g., C-O, O-C=O, and O-C(O)-O), which can decrease the surface energy of SnO_2_/rGO composites and form a superhydrophobic surface (Wu, et al., 2018; Xu, et al., 2019; Cao, et al., 2019). The performance of humidity insensitivity of the NO_2_ sensor may be weakened by working temperature (116°C) in some extent. However, the superhydrophobic structure of nanocomposites plays a vital role in the humidity insensitivity of the NO_2_ sensor (Phan and Chung, 2015; Wu, et al., 2018).

The relative response of the sensor increased along with the increase of the concentration of the NO_2_ whatever under humid air or not (50–1,500 ppb under dry air and 200–1,500 ppb under 70% RH) (Figure 4C). Meanwhile, it can be observed that the relative response exhibits a rapidly increasing linear trend under the low concentration of 1,000 ppb, indicating that SnO_2_/rGO composite-based sensors have an excellent sensing performance for the detection of low-concentration NO_2_. The observed slope differences between the low concentration (below 1,000 ppb) and the high concentration (1,500 ppb) could be attributed to the degradation of electron transfer and saturation phenomenon of the SnO_2_/rGO composites under high NO_2_ concentration. The linear fit of the gas response of the sensor to the various concentration of NO_2_ can be represented by relative response = *b* Concentration + *a*. The inset of Figure 4C indicates that the SnO_2_/rGO composite-based NO_2_ sensors have a linear correlation (*R* ^2^ > 98%). According to linear fit results, the theoretical limit of detection (LOD) of NO_2_ under 0 and 70% RH are calculated as 6.7 and 25 ppb based on Eq. 1, respectively, (Mateos, et al., 2019; Song, et al., 2016). Both LODs are below the threshold concentration for causing diseases and the NO_2_ pollutant concentration in the urban atmosphere.

$$LOD=3\times \frac{RMS_{noise}}{Slope}$$ (1)

**FIGURE 5:**
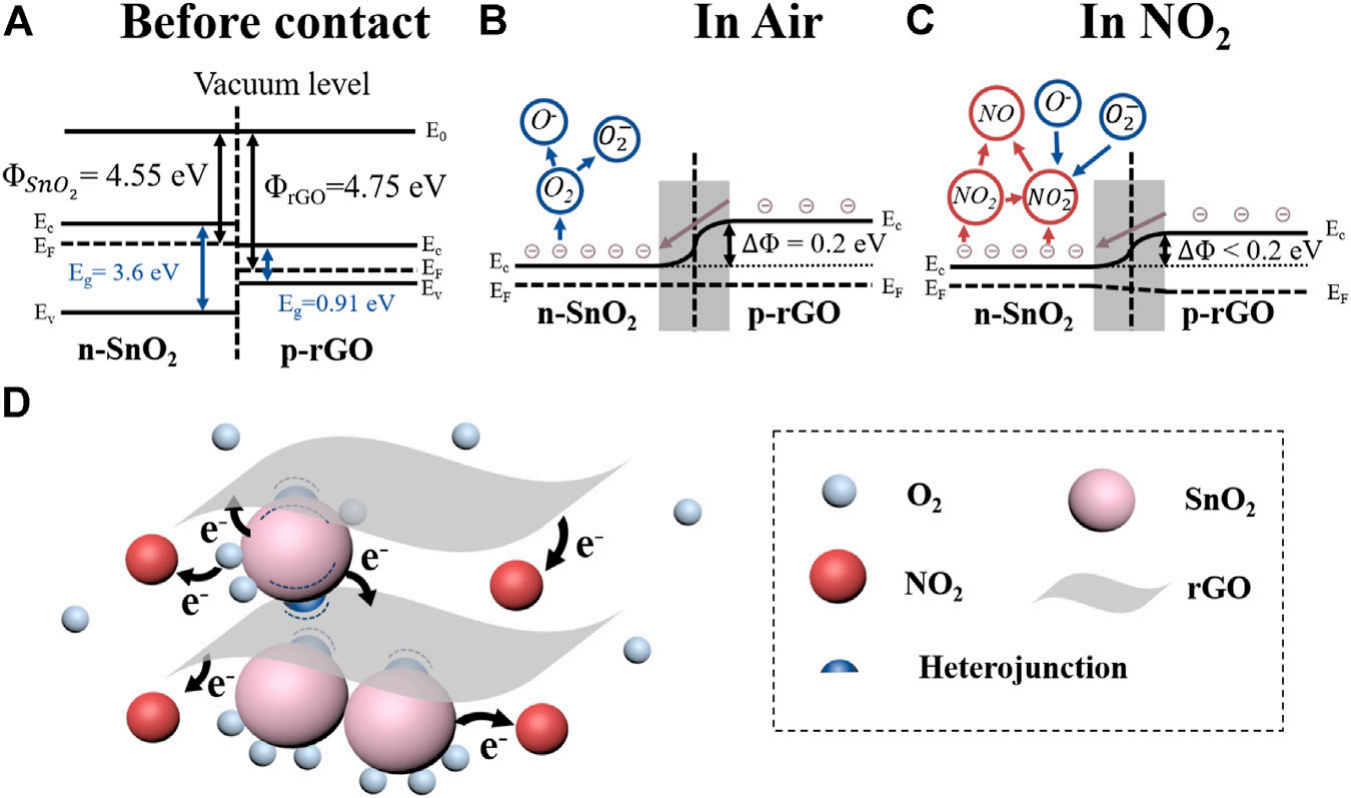
**(A)** Schematic bandgap structure of n-type SnO_2_ and p-type rGO; **(B)** Schematic bandgap structure of SnO_2_/rGO heterojunction in air and **(C)** in NO_2_; **(D)** Schematic of sensing mechanisms of SnO_2_/rGO composite-based sensors with exposure to NO_2_.

RMS_noise_ is the standard deviation of the noise level. Under dry air, the RMS_noise_ (0.029) was obtained from 150 baseline data points before exposure to NO_2_ from Figure 3D. Thus, according to Figure 5A (inset), the LOD was around 6.7 ppb. Under 70% RH, the *RMS*_*noise*_ (0.10) was obtained from 150 data. And the calculated LOD was about 25 ppb. Figure 4D illustrated the long-time stability of SnO_2_/rGO composite-based sensors to 800 ppb NO_2_ at room temperature for 28 days. The response is maintained between 8 and 9% and the standard deviation of NO_2_ sensors is 0.19, which indicated its good stability of sensitivity. However, the T90 and D10 varies as a function of deterioration of time which implies the sensor (Figure 4D inset).

### Sensing Mechanism

The sensing performance of SnO_2_/rGO composite-based sensors to NO_2_ shows a p-type sensing behavior indicating that the rGO dominates the conduction channel of NO_2_ sensors. In the vacuum state, the working functions of SnO_2_ (Φ_(SnO2)_) and rGO (Φ_(rGO)_) are 4.55 and 4.75 eV, respectively, (Kim, et al., 2016) (Figure 5A). As Figure 5B showed, after connection, the electrons will be transferred from SnO_2_ to rGO at the hetero-interface to balance the Fermi level (E_f_). Because of the charge transfer, it will form a depletion layer and heterojunction potential barrier (ΔΦ_hetero_ = 0.2 eV). In the air, oxygen molecules will adsorb on the surface of SnO_2_/rGO composites and withdraw electrons from SnO_2_ to form O_2_^−^ and O^−^. Since the diameter of SnO_2_ nanoparticles is comparable or less than two times the Debye length (*λ*_D_ ∼6 nm) of the SnO_2_ in the air, the SnO_2_ nanoparticles will almost fully deplete after adsorption of oxygen molecules (Xu, et al., 1991) (Figure 5B). Once exposed to NO_2_, owing to the high electron affinity of NO_2_ molecules, the NO_2_ molecules will further withdraw the electrons from oxygen ions and SnO_2_/rGO composites, as shown in Eqs. 2–5 and Figure 5C.

$$NO_{2}+e^{-}\to NO_{2}^{-}$$ (2)

$$NO_{2}+e^{-}\to NO+O^{-}$$ (3)

$$2NO_{2}+O_{2}^{-}+e^{-}\to 2NO_{2}^{-}+O_{2}$$ (4)

$$NO_{2}^{-}+O^{-}+2e^{-}\to NO+2O^{2-}$$ (5)

The Fermi level of SnO_2_ will shift far away from the conduction band leading to the reduction of the ΔΦ_hetero_. Meanwhile, the hole accumulation layer will form at the surface of rGO in the air and forma homojunction potential barrier (ΔΦ_homo_). When exposing to NO_2_, the NO_2_ molecules can directly adsorb on the surface of rGO and withdraw the electrons from rGO, which leads to an increasing in the hole concentration and reduction of the ΔΦ_homo_ (Miller, et al., 2014). As the resistance of sensors based on heterogeneous materials is exponential with the changing of the effective potential barrier (ΔΦ, including ΔΦ_hetero_ and ΔΦ_homo_, according to Eq. 6 (Feng, et al., 2017; Hua, et al., 2017). Thus, due to the synergistic effect of the ΔΦ_hetero_ and ΔΦ_homo_, the sensing performance of SnO_2_/rGO composites is greatly improved compared with bare rGO (Supplementary Figure S3).

$$R=R_{0}\text{exp}\left(\frac{\Delta \text{Φ}}{k_{b}T}\right)$$ (6)

where R_0_ is constant, k_b_ is Boltzmann’s constant, T is the absolute temperature, and ΔΦ is the effective potential barrier (including homojunction barrier and heterojunction barrier).

## Conclusion

In summary, this work demonstrated the simple sol-gel method to decorate rGO nanosheet with SnO_2_ NPs to improve NO_2_ detection. Due to the synergistic effect of SnO_2_/rGO p-n heterojunction and rGO/rGO homojunction, the sensing performance of SnO_2_/rGO composites was greatly enhanced compared with that of bare rGO. A low LOD of 6.7 ppb was obtained at 116°C under dry air. Compared with the reported humidity-insensitive NO_2_ sensors, the designed SnO_2_/rGO composite-based NO_2_ sensor showed an extremely high response ratio (649.0) between 750 ppb NO_2_ and 83% RH. The superhydrophobic property of the fabricated SnO_2_/rGO composites contributes to the humidity insensitivity. The superhydrophobic property is caused by the high roughness and low surface energy of the SnO_2_/rGO composites. It is promised to use the SnO_2_/rGO composite-based NO_2_ sensors in real-world applications.

## Data Availability

The original contributions presented in the study are included in the article/Supplementary Material, further inquiries can be directed to the corresponding authors.
