# Supplementary material for: Humidity-Insensitive NO2 Sensors Based on SnO2/rGO Composites
Source: Front Chem. 2021 May 28;9:681313. doi: 10.3389/fchem.2021.681313 (PMC8193670; doi:10.3389/fchem.2021.681313)
Supplement: Supplementary file 1 [file DataSheet1.PDF]

Supplementary Material

1 Supplementary Figures

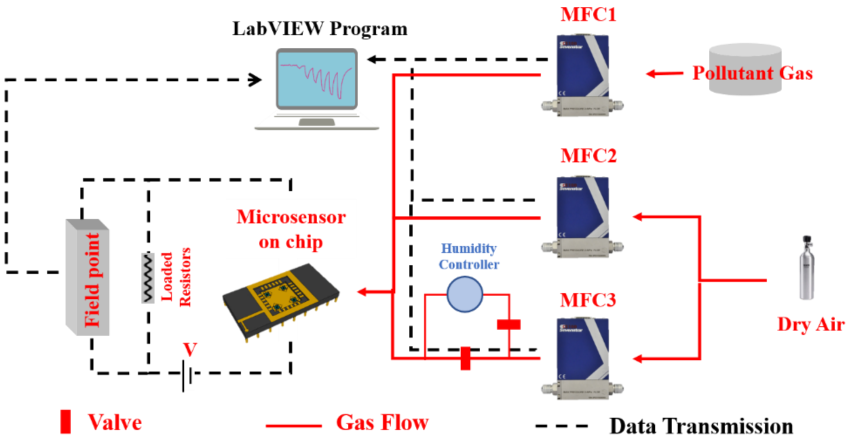

Figure S1. Schematic illustration of the gas testing system.

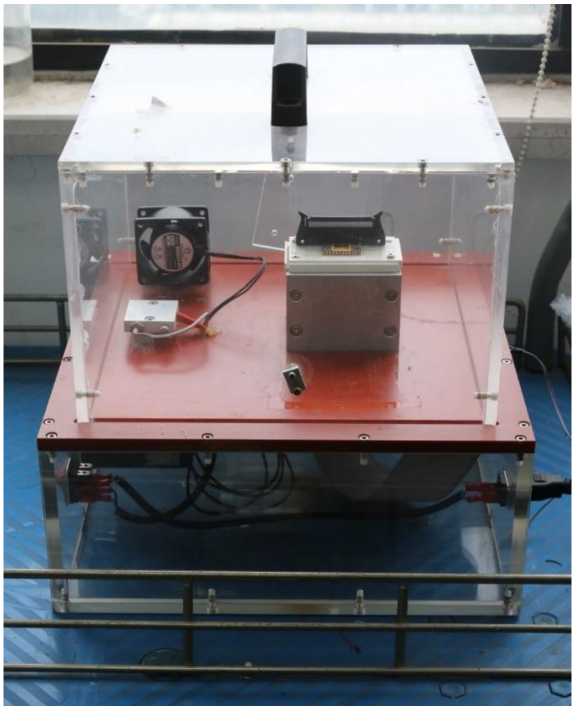

Figure S2. The photograph of the testing chamber we used to detect NO_2 in the real-world environment.

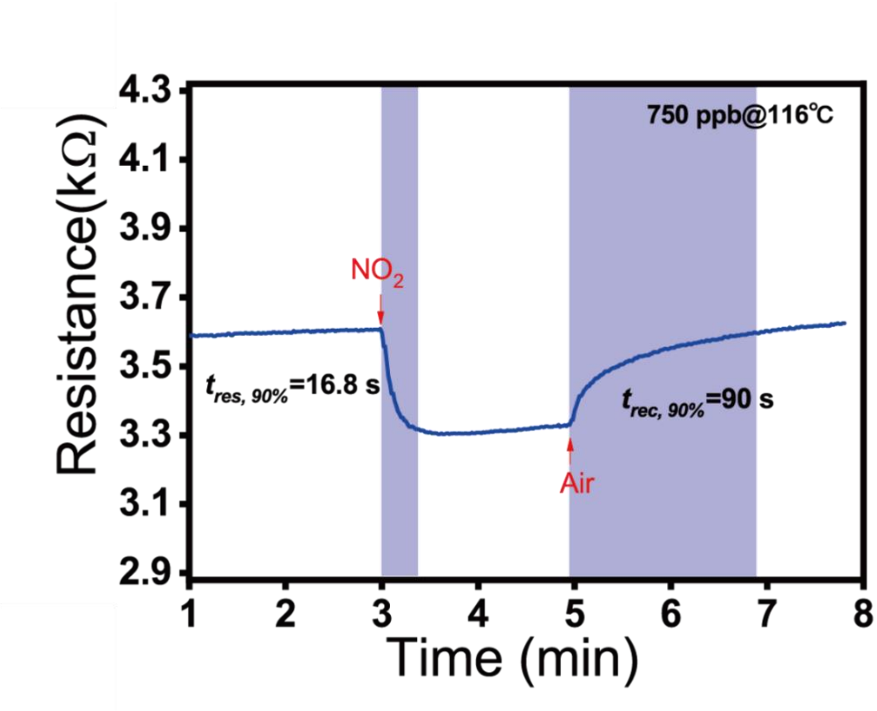

Figure S3. The variation of the measured resistance as a function of time during the sensing experiments.

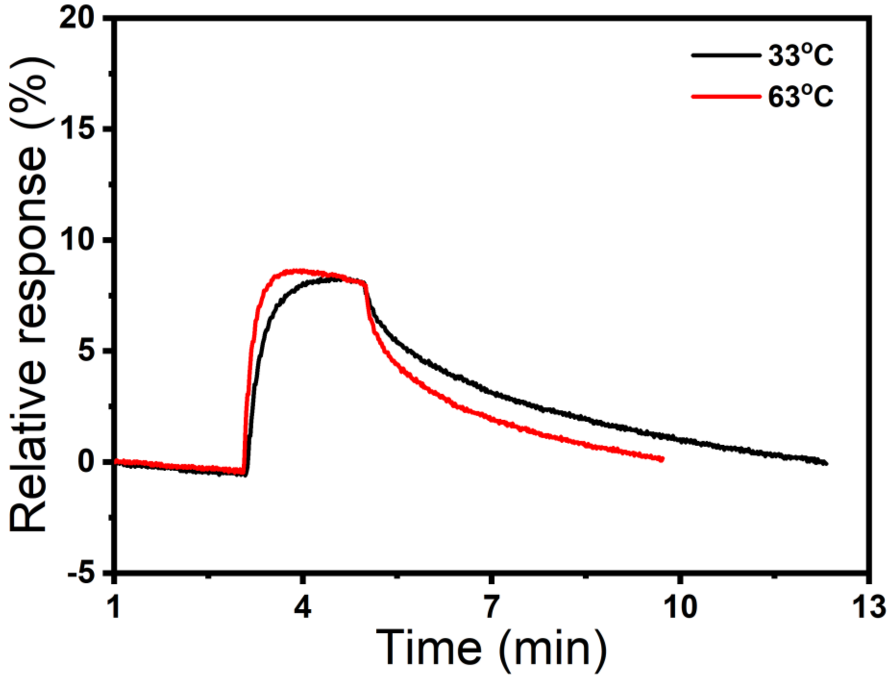

Figure S4. The curve of response time and recovery time of SnO_2/rGO sensor toward 750 ppb at 33℃ and 63℃.

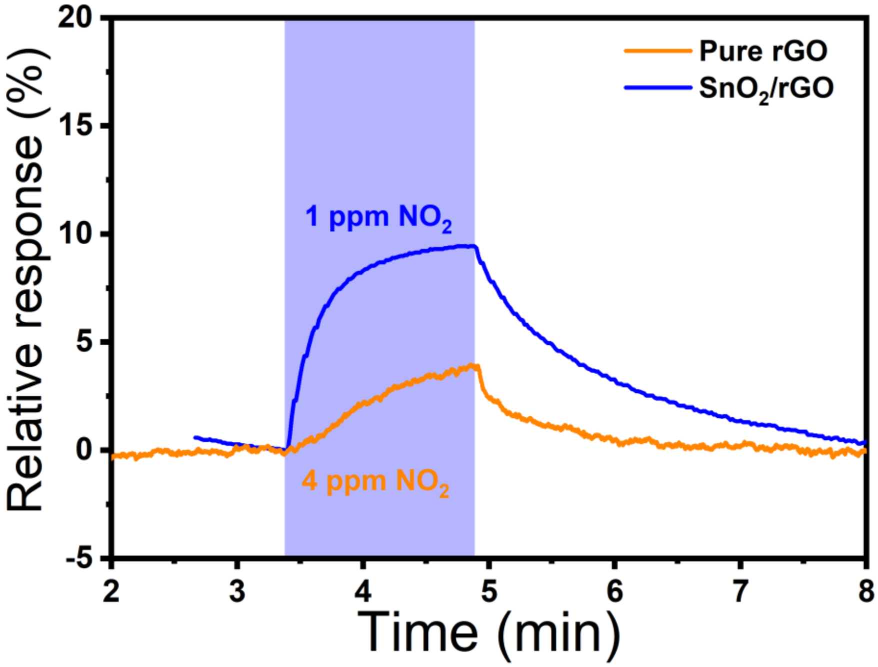

Figure S5. The comparison of sensing performance of the pure rGO (orange line) based sensor toward 4 ppm NO_2 and SnO_2/rGO (blue line) based sensor toward 1 ppm NO_2.

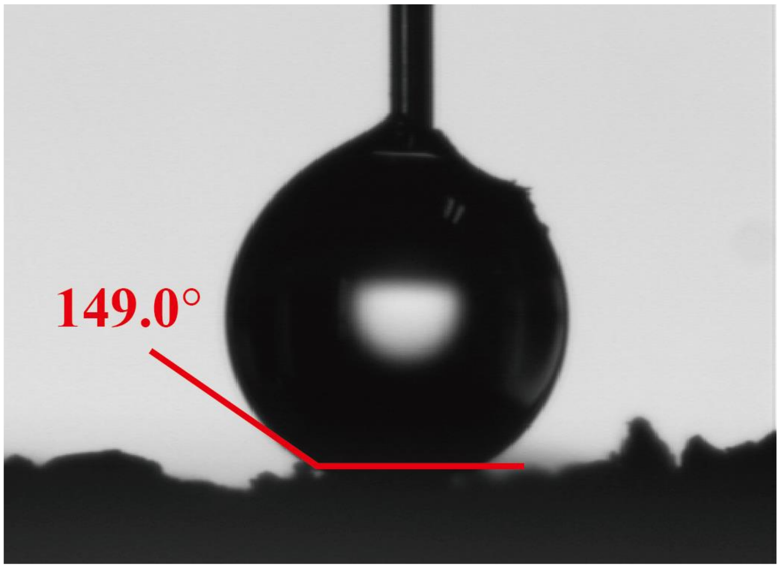

Figure S6. The contact angles of DI water droplets on SnO_2/rGO composites.

2 Supplementary Tables

Table S1. The adsorption energies of pollutant gases on graphene.

Pollutant gas	Adsorption energies (eV)
CO_2	-0.31 (Huang, et al., 2008)
NO_2	-2.7 (Huang, et al., 2008)
NH_3	-0.18 (Huang, et al., 2008)
Benzene	Nearly transparent (Chakradhar, et al., 2016)
Methanol	-0.22 (Schröder, 2013)
Toluene	-0.14 (Lazar, et al., 2013)

Table S2. The response value, response time, and recovery time of SnO_2/rGO sensor toward 750 ppb at various operating temperatures.

	33℃	63℃	116℃	189℃
Response value (%)	7.8	8.29	7.57	3.51
Response time (s)	36	24	17	7
Recovery time(s)	334	195	90	31

Table S3. Comparison of different sensing materials-based gas sensors in sensitivity and relative to humidity.

Materials	Target gas	Concentration (ppm)	Operation temperature (℃)	Relative Response (S_g, %) *	Response to relative humidity (S_{RH}, %)	Response Ratio (S_g/S_{RH}) **
rGO-CNT-SnO_2 hybrids (Liu, et al., 2015)	NO_2	5	RT	153	-	-
2H-WSe_2 (Moumen, et al., 2021)	NO_2	RT	RH	328%	-	-
Graphene with PS	NO_2	4.4	RT	~2.5%	8% (74%)	0.31

beads (Fei, et al., 2019)						
SnO_2/rGO (Zhang, et al., 2019)	H_2S	5	RT	~37	~25 (75%)	1.48
ZnO/SnO_2-RGO (Wang, et al., 2019)	NO_2	5	RT	141	~120 (75%)	1.17
SnO_2/N-RGO (Wang, et al., 2017)	NO_2	5	RT	38	4 (75%)	9.5
SnO_2/RGO hydrogel (Wu, et al., 2020)	NO_2	5	RT	32	5 (80%)	6.4
Commercial NO_2 sensor	NO_2	0.6	189℃	17.31	51.56 (83%)	0.36
SnO_2/rGO this work	NO_2	0.75	116℃	13.63	0.021 (83%)	649.0

*: Response is defined as $(R_g-R_a)/R_a$ or $(R_a-R_g)/R_a$

**: NO_2 to humidity response ratio between target gas and (~80% RH) humidity

3 References

Chakradhar A, Sivapragasam N, Nayakasinghe MT and Burghaus U. (2016). Adsorption kinetics of benzene on graphene: An ultrahigh vacuum study. *Journal of Vacuum Science & Technology A: Vacuum, Surfaces, and Films* 34, 021402. doi: 10.1116/1.4936337

Fei H, Wu G, Cheng WY, Yan W, Xu H, Zhang D, et al. (2019). Enhanced NO_2 sensing at room temperature with graphene via monodisperse polystyrene bead decoration. *ACS Omega* 4, 3812-3819. doi: 10.1021/acsomega.8b03540

Huang B, Li Z, Liu Z, Zhou G, Hao S, Wu J, et al. (2008). Adsorption of gas molecules on graphene nanoribbons and its implication for nanoscale molecule sensor. *The Journal of Physical Chemistry C* 112, 13442-13446. doi: 10.1021/jp8021024

Lazar P, Karlicky F, Jurecka P, Kocman Ms, Otyepková E, Šafářová Kr, et al. (2013). Adsorption of small organic molecules on graphene. *Journal of the American Chemical Society* 135, 6372-6377. doi: 10.1021/ja403162r

Liu S, Wang Z, Zhang Y, Zhang C and Zhang T. (2015). High performance room temperature NO_2 sensors based on reduced graphene oxide-multiwalled carbon nanotubes-tin oxide nanoparticles hybrids. *Sensors and Actuators B: Chemical* 211, 318-324. doi: 10.1016/j.snb.2015.01.127

Moumen A, Konar R, Zappa D, Teblum E, Perelshtein I, Lavi R, et al. (2021). Robust room-temperature NO_2 sensors from exfoliated 2D few-layered cvd-grown bulk tungsten di-selenide (2H-

WSe_2). *ACS Appl Mater Interfaces* 13, 4316-4329. doi: 10.1021/acsami.0c17924

Schröder E. (2013). Methanol adsorption on graphene. *Journal of Nanomaterials* 2013. doi: 10.1155/2013/871706

Wang Z, Gao S, Fei T, Liu S and Zhang T. (2019). Construction of ZnO/SnO_2 heterostructure on reduced graphene oxide for enhanced nitrogen dioxide sensitive performances at room temperature. *ACS Sensors* 4, 2048-2057. doi: 10.1021/acssensors.9b00648

Wang Z, Zhao C, Han T, Zhang Y, Liu S, Fei T, et al. (2017). High-performance reduced graphene oxide-based room-temperature NO_2 sensors: A combined surface modification of SnO_2 nanoparticles and nitrogen doping approach. *Sensors and Actuators B: Chemical* 242, 269-279. doi: 10.1016/j.snb.2016.10.101

Wu J, Wu Z, Ding H, Wei Y, Huang W, Yang X, et al. (2020). Three-dimensional graphene hydrogel decorated with SnO_2 for high-performance NO_2 sensing with enhanced immunity to humidity. *ACS Applied Materials & Interfaces* 12, 2634-2643. doi: 10.1021/acsami.9b18098

Zhang D, Wu Z and Zong X. (2019). Flexible and highly sensitive H_2S gas sensor based on in-situ polymerized SnO_2/rgo/PANI ternary nanocomposite with application in halitosis diagnosis. *Sensors and Actuators B: Chemical* 289, 32-41. doi: 10.1016/j.snb.2019.03.055
